# Supplementary material for: NPvis: An Interactive Visualizer of Peptidic Natural Product–MS/MS Matches
Source: Metabolites. 2022 Jul 29;12(8):706. doi: 10.3390/metabo12080706 (PMC9415073; doi:10.3390/metabo12080706)
Supplement: Supplementary file 1 [file metabolites-12-00706-s001.zip › metabolites-1796771-supplementary.pdf]

Supplementary Material for “NPvis: an Interactive Visualizer of Peptidic Natural Product–MS/MS Matches”

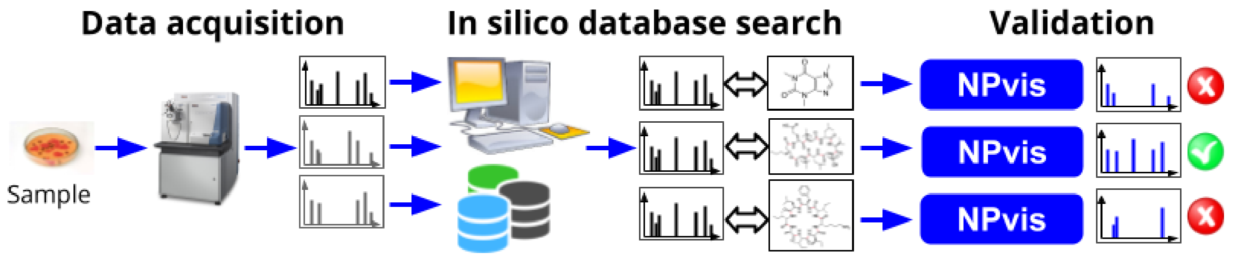

Supplementary Figure S1: Metabolomics in silico identification pipeline. Mass spectrometry analysis of a sample produces large amounts of metabolites tandem mass spectra (MS/MS). In this example, only three MS/MS are shown for simplicity. in silico identification methods coupled with chemical structure databases process MS/MS data and generate tentative annotations for some of the spectra. In this example, only one of the input spectra (shown in black) was annotated and there are three candidate chemical structures for it. NPvis could be used for annotation validation. In this example, the second annotation explains most of the MS/MS peaks, including the most intensive ones (shown in blue), so it is preferred over the two alternatives.

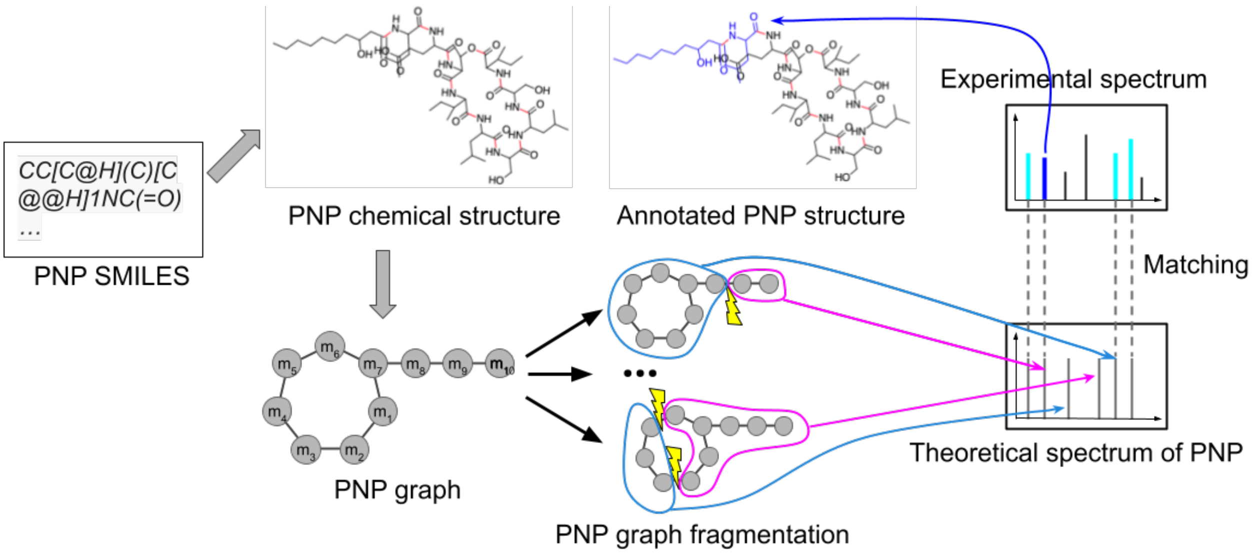

Supplementary Figure S2: The NPvis matching algorithm overview. An input peptidic natural product (PNP) in the SMILES format is converted into the chemical structure representation and the breakable bonds are identified (red). The structure is converted into a PNP graph with breakable bonds as edges and masses of the fragments between the bonds as nodes. NPvis models the MS/MS fragmentation by breaking every single edge and pair of edges in the PNP graph that lead to the graph decomposition into two components. The masses of the components are considered as theoretical peaks that altogether form the theoretical spectrum of the PNP. NPvis compares the theoretical spectrum with its experimental counterpart and considers an observed MS/MS peak annotated by a PNP fragment if their masses are within a small user-configurable threshold. As an example, here the mass of the blue-colored fragment in the PNP structure matches the mass of the second peak in the experimental spectrum (blue).

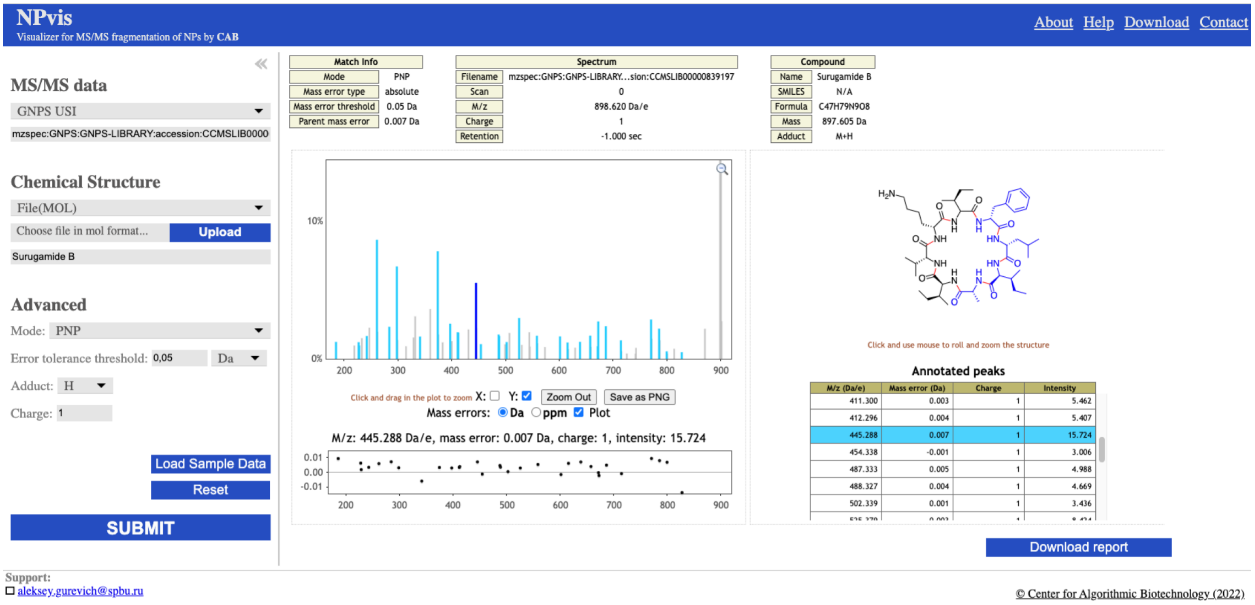

Supplementary Figure S3: The NPvis web server visualisation of surugamide B mass spectrum. The spectrum is provided via GNPS USI, the structure is provided as a MOL file. NPvis was run in the "PNP" mode, so the exact matching was performed and modification information is not shown. The maximum allowed mass error was set to 0.05 Da, but the error plot shows that all peaks were annotated even within 0.01 Da tolerance. The MS/MS plot is zoomed to the 10% intensity (the Y-axis) since all peaks are below this level except the one corresponding to the entire molecule (m/z 898.6).

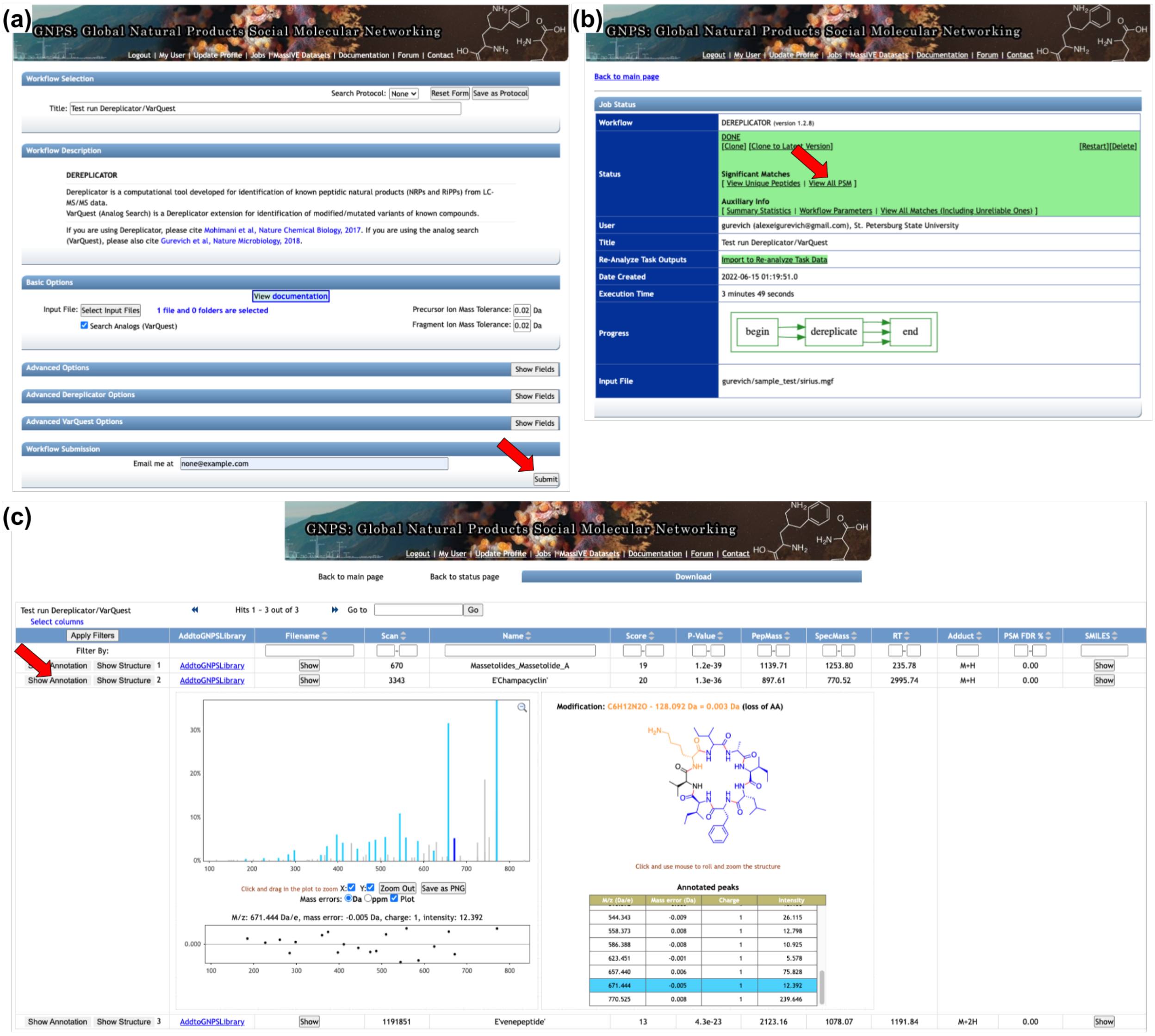

Supplementary Figure S4: The NPvis integration with GNPS. (**a**) The Derelicator/VarQuest workflow at the GNPS platform. Users select input spectra files, adjust the workflow parameters, press the Submit button (red arrow), and wait for the data analysis results. (**b**) The job status page of the completed Derelicator/VarQuest job. Users may view the most reliable in silico spectra annotations by clicking on the View All PSMs link (red arrow). (**c**) The list of all identified Peptide-Spectrum Matches (PSMs). Each row contains the Show Annotation button (red arrow) that opens NPvis visualisation of the corresponding match. The demonstrated workflow results are available from `https://gnps.ucsd.edu/ProteoSAFe/status.jsp?task=49e24d19c2b24bc7a82cd65319ce91c0` (accessed on 28 July 2022).
